# Advances in the Study of Structural Modification and Biological Activities of Anoplin

**DOI:** 10.3389/fchem.2020.00519

**Published:** 2020-07-07

**Authors:** Ye Wu, Rui Huang, Jin-Mei Jin, Li-Jun Zhang, Hong Zhang, Hong-Zhuan Chen, Li-Li Chen, Xin Luan

**Affiliations:** Institute of Interdisciplinary Integrative Medicine Research, Shanghai University of Traditional Chinese Medicine, Shanghai, China

**Keywords:** anoplin, structural modification, structure–activity relationship, antimicrobial activity, antitumor activity

## Abstract

Anoplin is an amphipathic, α-helical bioactive peptide from wasp venom. In recent years, pharmaceutical and organic chemists discovered that anoplin and its derivatives showed multiple pharmacological activities in antibacterial, antitumor, antifungal, and antimalarial activities. Owing to the simple and unique structure and diverse biological activities, anoplin has attracted considerable research interests. This review highlights the advances in structural modification, biological activities, and the outlook of anoplin in order to provide a basis for new drug design and delivery.

## Introduction

Wasp venom is the light yellow, transparent liquid secreted by the venom glands and accessory glands of wasp; it is stored in a poison sac and discharged by sting (Monteiro et al., 2009). In general, venom is used as a protective measure against the invaders that threaten their survival or colony (Hou et al., 2014). It is worth mentioning that, for wasps, venom is also used during the hunting of prey (Yan et al., 2011). Wasp venom is mainly composed of a few biological active peptides (like mast cell degranulating peptides and mastoparan) (Argiolas and Pisano, 1985; Monteiro et al., 2009), active enzymes (phospholipase A2 and hyaluronidase) (Tsai et al., 2011), histamine, 5-hydroxytryptamine, choline, glycerin, amino acids, and so on (Schmidt, 1982). Among them, peptides in the wasp venom usually exhibit superior pharmacological effects and a promising clinical value (Arifuzzaman et al., 2019; Li et al., 2019).

**Figure 1:**
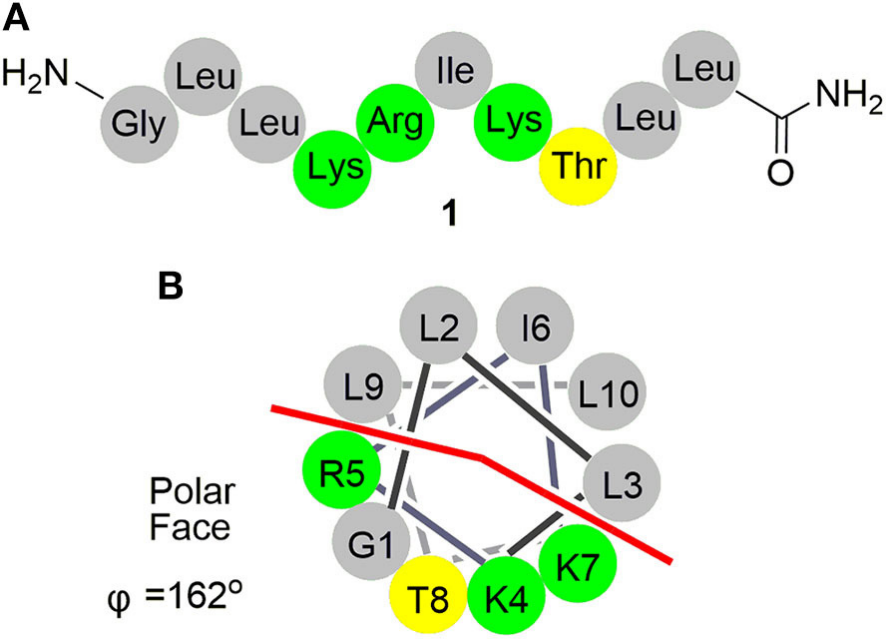
**(A)** Schematic representation of anoplin (**1**). **(B)** Helical wheel diagram of anoplin showing the polar and nonpolar faces of the helix. All residues are drawn in helical configuration. Gray symbols represent hydrophobic residues. Yellow symbols represent polar, uncharged residues. Green symbols represent basic residues.

As a wasp venom peptide, anoplin (**1**, Figure 1) was isolated from the venom sac of the Japanese solitary spider wasp *Anoplius samariensis* (Hisada et al., 2000). Anoplin is the shortest, amphipathic, linear α-helical antimicrobial peptide (AMP) with only 10 residues (Gly-Leu-Leu-Lys-Arg-Ile-Lys-Thr-Leu-Leu-NH_2_) (Konno et al., 2001; Jittikoon, 2015); it also exhibits a wide range of biological activities including antibacterial (Konno et al., 2001; Monincová et al., 2010), mast cell degranulating (Cabrera et al., 2009), antitumor (Zhu et al., 2013; Da Silva et al., 2018; Kai et al., 2018), antimalarial (Carter et al., 2013), antifungal (Jindrichova et al., 2014), and anti-inflammatory activities (Zhong et al., 2020b). Anoplin exerts its functions by direct interaction with anionic bilayers and biological membranes via ion channels (Cabrera et al., 2008; Leung et al., 2011), selectively binding to the bacterial DNA or inhibiting ATP synthase (Syed et al., 2018). Owing to its extremely simple structure and nonhemolytic toxicity, anoplin exhibits superiority in chemical manipulation, structure–activity relationship studies, mechanisms of action, and medical application, which has a great potential as a novel class of drugs for antibiotics and anticancer applications. Given the fact that there is no report on research progress about structural modification of anoplin so far, we have summarized the advances in structural modification and relevant pharmaceutical activities to push the further application of anoplin.

## Structural Modification of Anoplin

### Point Mutation and Truncation

The Hansen lab (Ifrah et al., 2005) firstly conducted the structure–function and structure–toxicity relationships research of anoplin by point mutation, C- and N-terminal truncation. Terminus truncations indicated that the full length of decapeptide is necessary for antibacterial activity toward *Escherichia coli* or *Staphylococcus aureus*. Then standard Ala-scan revealed Gly-1, Leu-2, Leu-3, Ile-6, Leu-9, and Leu-10 are critical residues. A series of studies of analogs with different residue substitutions showed that hydrophobic anoplin derivatives generally have a lower minimum inhibitory concentration (MIC). On the other hand, it will also have higher hemolytic activity and reduce or reverse the selectivity between *E. coli* and *S. aureus*. More importantly, the overall charge of +4 is essential for anoplin to differentiate bacteria and normal cells.

**Figure 2:**
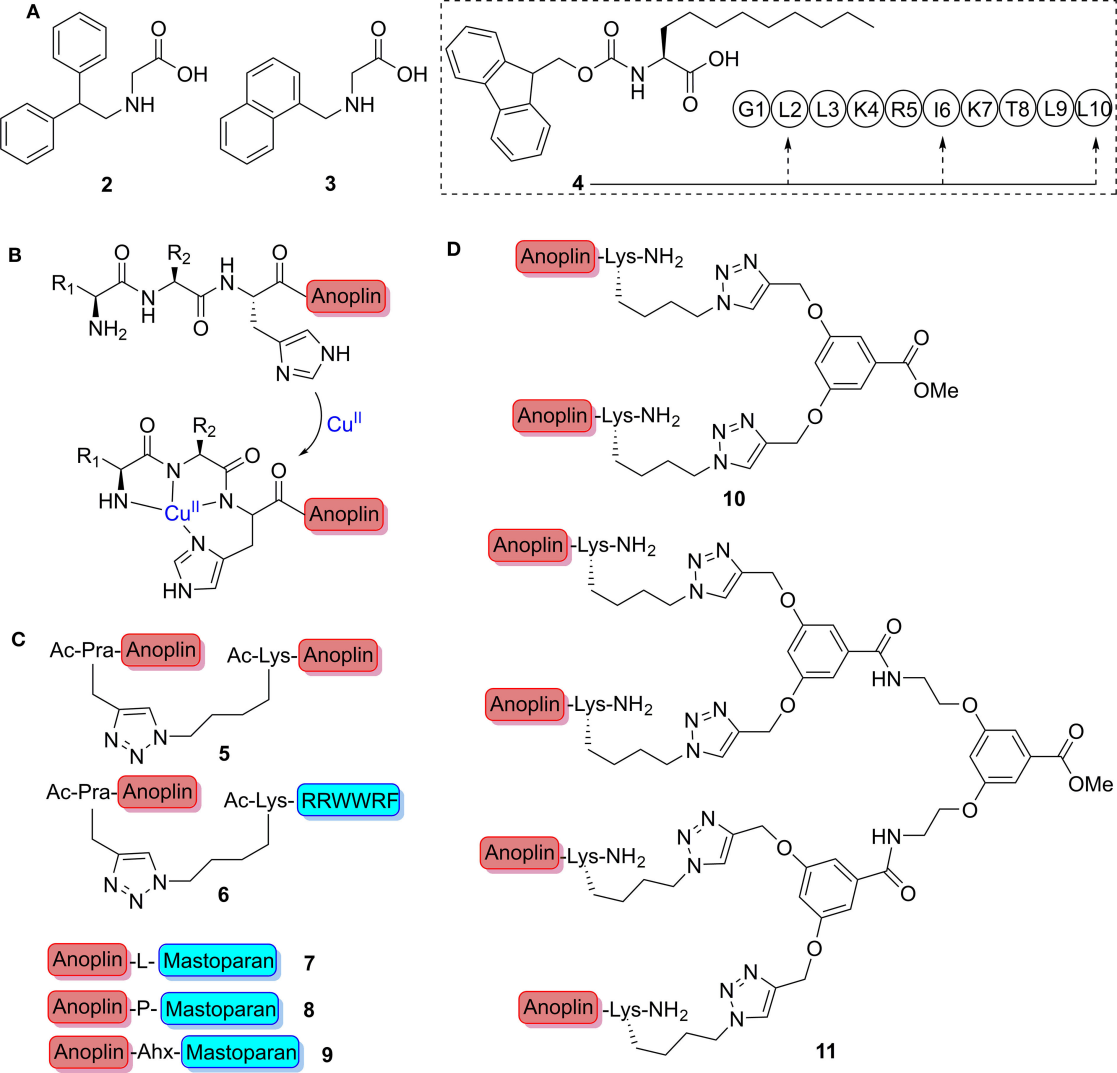
**(A)** Point mutation of nonnatural amino acid. **(B)** Schematic representation of the ATCUN-anoplin bound to a Cu^2+^ ion. **(C)** Structure of dimeric peptides. **(D)** The conjugation of anoplin to divalent and tetravalent dendrimers.

After Meinike et al. verified the substitution of Arg-5 with different hydrophobic amino acids that resulted in potent analogs, they further replaced Arg-5 with peptoid monomers. Among the tests, the therapeutic index (TI; larger values indicate an improved activity specificity) of analogs containing *N*-(2,2-diphenylethyl) Gly or *N*-(1-naphthalenemethyl) Gly (**2** or **3**, Figure 2A) is commendably comparable with that of anoplin, but the selectivity was reversed toward *E. coli* and *S. aureus* (Meinike and Hansen, 2009).

To further explore suggested activity-improving discoveries, the Hansen group (Munk et al., 2013) presented 19 new anoplin analogs that substituted in amino acid positions 2, 3, 5, 6, 8, 9, and 10 with Lys, Trp, Phe, β-2-naphthylalanine (2Nal), and β-cyclohexylalanine (Cha). Some discrete substitutions using Lys showed some promise, but Trp and Phe did not provide anoplin analogs with improved microbial specificity or lessened hemolytic activity (Braun and Heijne, 1999; Chen et al., 2005; Schmitt et al., 2007; Wiradharma et al., 2011). This was probably because Trp and Phe in those sites hindered the interaction between membrane and peptides owing to the structural distortions. It was gratifying that the use of 2Nal^6^ and Cha^3^ exhibited promising TI against all strains tested. Afterwards, they successfully developed analysis of variance models that describe the relationship between peptide properties (high-performance liquid chromatography retention time, hemolytic property, and MIC) and the structural characteristics of the anoplin analogs by point mutation, and they verified the properties predicted by the mathematical models, which were in reasonable agreement with the measurements (Munk et al., 2014).

Cabrera et al. (2008) synthesized deamidated analog of anoplin to explore the effect of deamidation at the C-terminus, and they found that although anoplin-OH still retained the same α-helical content, it lost the characteristic antimicrobial and mast cell degranulating activities of anoplin. A reasonable explanation was that carboxylation correlated with electrostatic repulsion and amphipathic character. Subsequently, Pripotnev et al. confirmed that C-terminal amide group was important for the structural stabilization, extra charge and function of anoplin, and the physicochemical properties of peptides defined its AMP activity (Pripotnev et al., 2010). However, the amidation of anoplin was not essential for its antifungal and plant defense stimulating activities (Jindrichova et al., 2014).

Won et al. (2011b) applied several biophysical techniques (UV resonance Raman spectroscopy in combination with Langmuir–Blodgett monolayer technique vesicle leakage assay) to study the relationship between physicochemical properties (secondary structure and surface activity) with biological activities of anoplin and its two derivatives-anoplin-8K (GLLKRIKKLL-NH_2_) and anoplin-1K5V8K (KLLKVIKKLL-NH_2_). These results indicated that higher helical tendency, stronger amphipathicity, and extra positive charges could be beneficial for higher antimicrobial activity with weak membrane lytic activity.

As a membrane anchor, the length of lipophilic alkyl chain of lipopeptides is considered highly vital for the bioactivity of peptides (Taft and Selitrennikoff, 1990). To increase the membrane affinity of anoplin without sacrificing important positively charged side chains, Slootweg et al. incorporated the lipophilic amino acid, (*S*)-2-aminoundecanoic acid (**4**, Figure 2A), which they synthesized into the peptide sequence (Leu-2, Ile-6, and Leu-10) of anoplin (Slootweg et al., 2013). As expected, all the derivatives exhibited enhanced activities and selectivity toward microbial membranes, while the effect of hemolysis showed considerably increase.

To better comprehend the structure–activity relationships of short cationic α-helical AMPs, and to achieve a generally applicable set of guidelines on how to increase AMP activity without concomitant introduction of strong hemolytic activity, Wimmer and the Hansen group (Uggerhoj et al., 2015) presented the NMR structure of anoplin in a micellar environment and systematically set up a vast library of substitutions for antimicrobial activity, hemolytic activity, and changes in structure and lipid interactions. They successfully proposed a do's and don'ts list with the core concept of subtle hydrophobicity increase in the hydrophobic face and the polarity increase in the hydrophilic face, including the following: (1) do not rely on the helical wheel model to design AMP derivatives, especially for nonconservative point mutations; (2) do identify whether each residue interacts with the membrane interior, with lipid head groups, or with the bulk solvent; (3) do not substitute those hydrophobic residues that interact with the lipid head; (4) do increase the polarity of side chains that interact with bulk solvent; and (5) do increase the hydrophobicity of the hydrophobic face in small steps and a few positions. Based on these findings, it is not hard to see that the stronger amphipathicity plays an important role for the activity of anoplin.

### N-Terminal Auxiliary

Amino-terminal copper and nickel (ATCUN) binding motifs, H_2_N-AA_1_-AA_2_-His, are known to actively form reactive oxygen species (ROS) upon metal ions binding, and ROS is primarily a result of Cu(II) binding (Harford and Sarkar, 1997; Donaldson et al., 2001; Du et al., 2013) (Figure 2B). The formed ROS can render the bacteria more susceptibility to antimicrobial agents (Fang, 2004). The Angeles-Boza group (Libardo et al., 2014) prepared three ATCUN binding motifs containing derivatives of anoplin (Asp-Ala-His, Gly-Gly-His, and Val-Ile-His), which were found to be more active than the parent anoplin against a panel of clinically relevant bacteria with non-hemolysis through ROS-induced membrane damage. Whereafter, they found two additional ATCUN sequences (Arg-Thr-His and Leu-Lys-His), which produce ·OH and other ROS at faster rates than did other ATCUN complexes. When evaluated by the degree of oxidative stress brought induced by new ATCUN-anoplin conjugates, the Arg-Thr-His-anoplin peptide was up to four times more potent than is anoplin alone against standard test bacteria (Libardo et al., 2015). The metal binding tripeptide motifs provided a simple approach to increase potency of anoplin by conferring a secondary action.

Similar with lipophilic alkyl chain of lipopeptides, acylation of the N-terminus with fatty acid moiety can also increase the membrane affinity and the activity of AMPs (Avrahami and Shai, 2002). So Chionis et al. (2016) incorporated the lipophilic octanoic, decanoic, and dodecanoic acid residues into the N-terminus of anoplin. Those results indicated that fatty acid moiety was good for the membrane affinity, antimicrobial potency, and the higher helical content of the peptides, and these peptides were nontoxic to erythrocytes and stable to proteolysis. In addition, the increase of the length of the lipophilic moiety did not further affect the above properties. Salas et al. (2018) thought that the loss of activity upon truncation of anoplin in the literature (Ifrah et al., 2005) was correlated to lowering of hydrophobicity, so they synthesized and investigated the effects of truncated palmitoylated anoplin analogs, and their results demonstrated that palmitoylation of truncated anoplin analogs can increase antimicrobial activity, and even helicity, in water.

### d-Amino Acid Conversion

d-Peptide and d-protein composed of d-amino acids are potential therapeutic agents with longer half-lives or oral possibility *in vivo* (He et al., 1996; Weinstock et al., 2014). Therefore, d-amino acid substitution is a useful method to improve the enzymatic stability and activity of AMPs. Won et al. (2011a) found that the replacement of all amino acids with their d-stereoisomers did not change bactericidal activity of anoplin, so the antibacterial mechanism is through nonspecific interaction.

Inspired by the experience of Ifrah et al. (2005), Wang et al. (2014) designed and synthesized a group of anoplin analogs by multiple residue substitutions with d-amino acids. Owing to the increased charge, hydrophobicity, and amphiphilicity, anoplin-4 (kllkwwkkll-NH_2_) composed of d-amino acids displayed the characteristics of the highest antimicrobial activity *in vivo*, higher proteolytic stability, and lower toxicity to normal cells, which make anoplin-4 a great candidate for future optimization and treatment of infection.

### Dimerization and Multivalent Presentation

Hybrid peptide analogs can effectively combine the therapeutic advantages of different peptides and achieve collaborative treatments (Liu et al., 2013; Kamysz et al., 2015; Le et al., 2015). Hexapeptide, RRWWRF (named RW), is a cationic peptide derived from the screening of a hexapeptide combinatorial library; it is beneficial to interact with the negatively charged components of the bacterial membrane (Haug et al., 2008). Liu et al. (2017) successfully synthesized the homodimeric peptide **5** and the heterodimeric peptide **6** by copper(I) catalyzed azide-alkyne cycloaddition (CuAAC “click chemistry”) (Figure 2C); the dimer peptides not only had significantly enhanced antimicrobial activity against multidrug-resistant (MDR) bacteria *in vitro* and *in vivo* but also showed synergy and additivity effects when used in combination with conventional antibiotics rifampin or penicillin.

To break α-helix and obtain greater flexibility of heterodimeric AMPs, Kai et al. (2018) used different amino acids (**7–9**, Figure 2C), including Leu, Pro, and Ahx (aminocaproic acid), as linker to connect anoplin and mastoparan, another α-helical AMP from *Vespula lewisii* venom. Findings manifested that Pro and Ahx contributed to the design of ideal dimer AMPs with high selectivity and potency while reducing lytic activity with respect to red blood cells. However, Leu cannot confer increased flexibility to heterodimeric AMPs. It is flexible heterodimeric structure induced by Pro and Ahx that improved the potency of heterodimeric AMPs for negatively charged membranes while reducing interaction with zwitterionic membranes. As can be seen, dimerization offers an effective strategy to screen for the development of novel antimicrobial agents.

Given the toxicity, the short half-life *in vivo*, and stimulation of an immune response of AMPs, multivalent AMPs formed by attaching several peptide monomers to reactive polymer scaffolds via naturally occurring intermolecular disulfide bridges or unnatural scaffold linkers have been used to overcome the problems mentioned above (Liu et al., 2010). Chamorro et al. showed that anoplin was successfully conjugated via click chemistry to divalent and tetravalent dendrimers (**10** and **11**, Figure 2D) (Chamorro et al., 2012). Compounds **10** and **11** clearly resulted in enhanced pore formation of anoplin.

**Figure 3:**
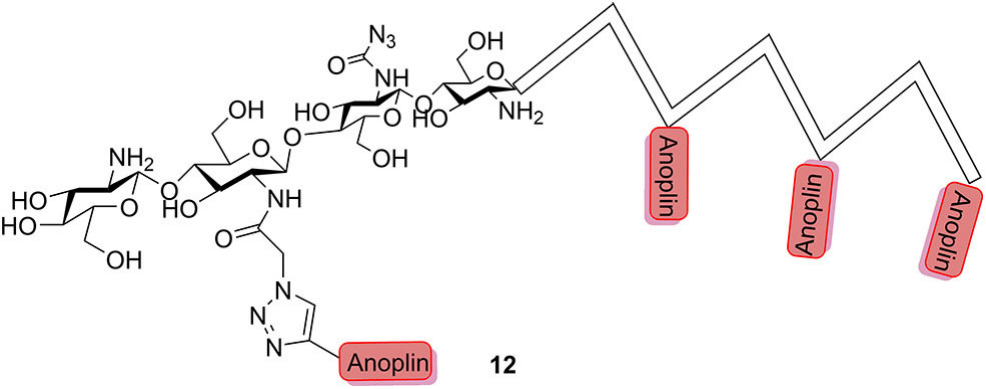
Schematic representation of anoplin–chitosan conjugates.

Chitosan is a natural polymer comprised of β-(1-4)-linked glucosamine and some degree of *N*-acetyl glucosamine with low antimicrobial activity and has been widely applied in the field of biomedicine. Sahariah et al. (2015) reported that grafting of anoplin to chitosan polymers was a rational design for abolishing the hemolytic propensity and increasing the activity of the parent peptide; at the same time, the anoplin–chitosan conjugates had a high degree of control over the resulting peptide grafting density (**12**, Figure 3).

### Combined Modification

**Figure 4:**
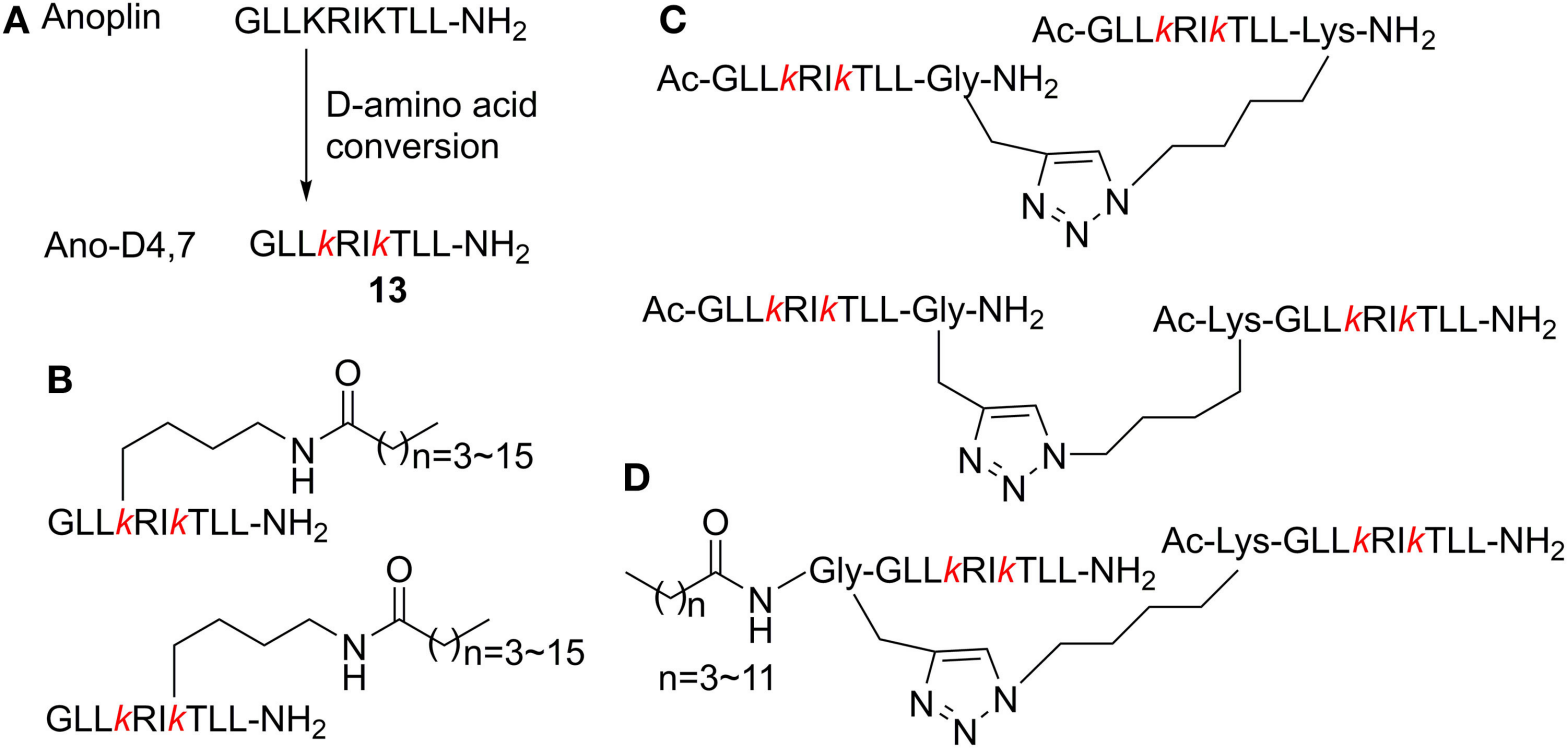
**(A)** d-Amino acid conversion of anoplin to Ano-D4, 7. **(B)** Antimicrobial peptides conjugated with fatty acids on the side chain of Ano-D4, 7. **(C)** Different dimerization positions based on Ano-D4, 7. **(D)** N-terminal fatty acid modified-dimer Ano-D4, 7.

Combination of multiple modification methods may represent more effective strategies with their respective advantages. Based on the substitutive anoplin sequence Ano-D4, 7 (**13**, Figure 4A), GLLkRIkTLL-NH_2_ (lowercase letters indicate the d-enantiomer), further modifications were carried out to systematically screen excellent AMPs with optimal therapy potential. Zhong et al. (2020b) conjugated various lengths of fatty acid chains ranging from 4 to 16 carbons onto the side chain of the position 4 or 7 d-amino acid of Ano-D4, 7 (Figure 4B). The antimicrobial activity of those new peptides was highly correlated with the lengths of the fatty acid chain. When the length of the fatty acid chain was ≤12 carbons, there was a remarkable increase in the MIC values of the new peptides after the hydrophobicity went beyond preceding threshold. Interestingly, the peptides conjugated with the same fatty acid chain (≤12 carbons) at position 7 of Ano-D4, 7, exhibited better activity than the corresponding peptide with the modification at position 4. This is probably because side chains of Arg-5 and Thr-8 shielded the fatty acid on the position 4 d-Lys side chain. These peptides also significantly reduced the bacterial load in mice without obvious adverse reactions or any death at the effective dose.

According to previous studies on dimeric modification, Zhong et al. (2020a) further inquired the effects of different dimerization positions on biological activity. They found that the anti-biofilm activity of the C- and N-terminal dimer peptides was better than C–C terminal ones (Figure 4C). Meanwhile, new peptides combining d-amino acid conversion and dimerization displayed higher TI without hemolytic activity. In addition, on the basis of the previous two reports, they designed dimer peptide conjugated fatty acids at the N-terminus of Ano-D4, 7 and dimerization (Figure 4D) (Zhong et al., 2019). The multi-modified analogs were more inclined to present β-strand structure; this pre-assembled state of the dimer peptides led to fast interaction without the demand for extra aggregation process, which caused more efficient permeabilization. Furthermore, the previous peptides also exerted high stability toward protease, serum, salts, and different pH environments, which proved that combined modification techniques provided more flexible approaches to develop novel AMPs.

## Potential Development Direction of Anoplin

### Conformational Constraint

**Figure 5:**
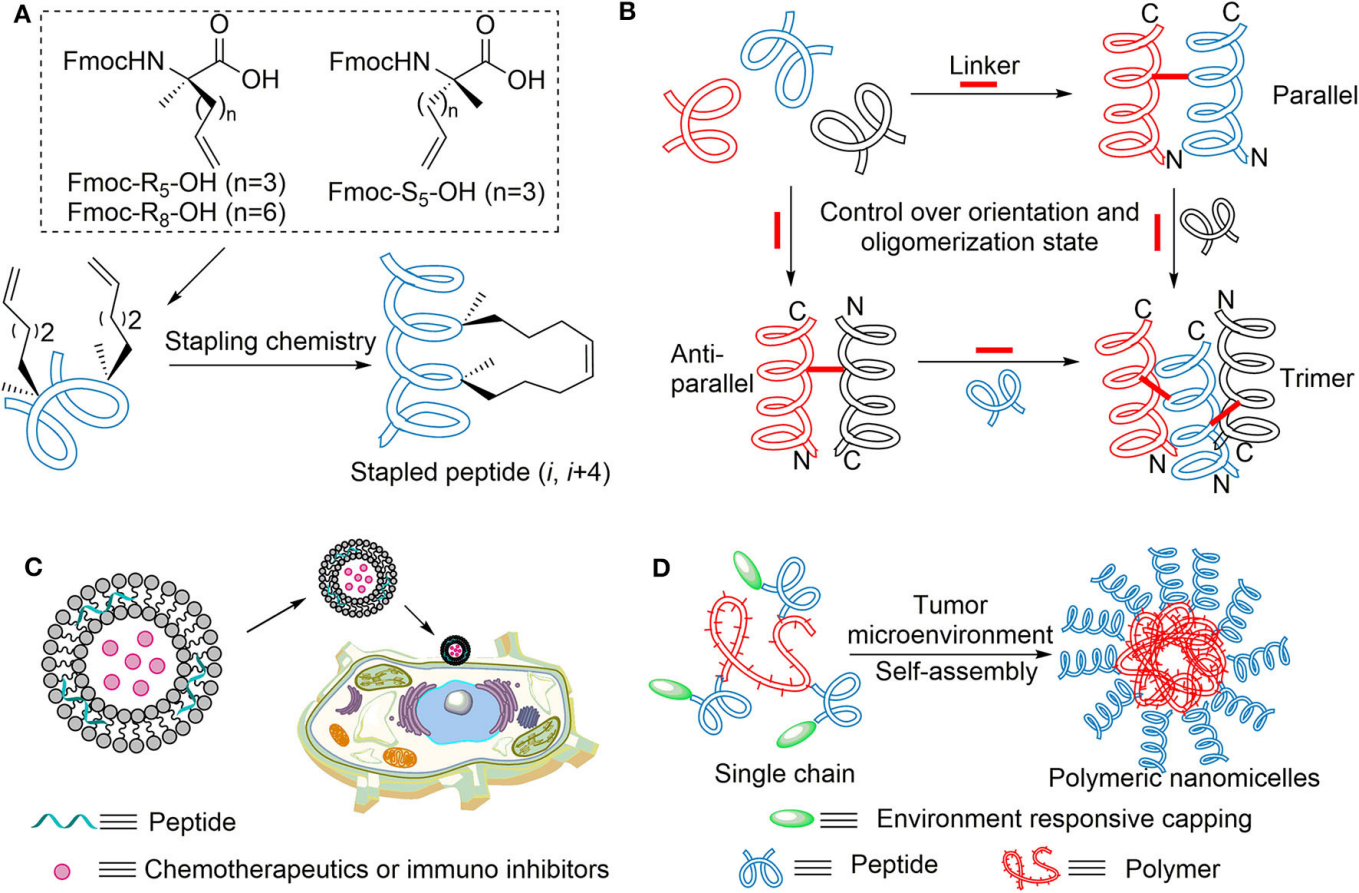
**(A)** All-hydrocarbon stapled peptide strategy. **(B)** Strategies to induce helix dimer and trimer formation of designed peptides. N and C designations indicate the amino and carboxy termini in the helix models, respectively. **(C)** Targeted delivery system based on bioactive peptides. **(D)** Self-assembly of polymer–peptide conjugates in tumor microenvironment.

Despite the above notable advances, there are still many disadvantages in the previous modifications. For example, residue substitutions cannot solve the low stability toward protease, and there also may be metabolic problems for d-peptide *in vivo* compared with natural amino acid. The synthesis of dimerization and multivalent presentation are difficult and tedious. A promising alternative approach would constrain α-helical conformation by stapling chemistry (Schafmeister et al., 2000; Walensky et al., 2004; Moellering et al., 2009; Walensky and Bird, 2014) (Figure 5A). The first stapled peptide, ALRN-6924, is currently in phase II trials to treat advanced solid tumors or lymphomas in connection with MDM2 and MDMX (Carvajal et al., 2018; Ng et al., 2018). We have previously reported the design and synthesis of a series of hydrocarbon stapled melittin peptides of which, some analogs showed remarkable enhancement not merely in antihepatoma activity but also in α-helicity and protease resistance (Wu et al., 2017a). Subsequently, we developed a new series of stapling amino acids, which contained the native amino acid side chains to expand the scope of the all-hydrocarbon stapled peptide strategy (Wu et al., 2017b).

In addition to α-helical constraint of single short peptide, nature actually exhibits its own approach to maintain the structural integrity of proteins and protect natural peptides from enzymolysis. A noteworthy example was the self-assembly of β-sheet peptides into amyloid-β protein by inter-strand hydrophobic interactions, which exhibited extreme resistance toward intracellular proteases (Barz et al., 2018). Inspired by this, the Wu group reported that crosslinked helix dimers can be constructed by two disulfide bonds for ultrahigh proteolytic stability (Chen et al., 2015). To overcome the instability of disulfide linkage under the intracellular reducing condition, Wuo et al. developed more judicious covalent bonds like bis-triazole linkers (Wuo et al., 2015) and hexafluoroisopropanol-based bisthioether crosslinkers (Wuo et al., 2018; Chen et al., 2019) (Figure 5B). We expect these effective strategies be used to modify anoplin with preferable biological function.

### Targeted Delivery System

Bio-functional nanosystems are also regarded as novel strategies to address some drawbacks of peptides (Aronson et al., 2018). Through the widely accepted enhanced permeability and retention (ERP) effect (He J. Y. et al., 2019), drug delivery platforms based on cytotoxic peptides possessed prolonged circulation time and targeting capability, which improve its therapeutic utility and reduce side effects. More importantly, this technology can achieve co-delivery of multiple drugs for the purpose of combination therapy in precision medicine (Jin et al., 2018) (Figure 5C). To obtain deeper solid-tumor penetration and perfuse more homogeneously within tumor tissue, one ingenious design is tumor microenvironment (TME)-induced *in situ* self-assembly of polymer–peptide conjugates (Cong et al., 2019) (Figure 5D); this transformation-enhanced accumulation and retention (TEAR) effect showed higher anticancer activity than the ERP effect of traditional nanoparticles (He P. P. et al., 2019).

## Conclusion and Outlook

The emergence of MDR bacteria and the markedly declining supply of safe and efficacious antibiotics have made it urgent to develop new antibiotics. On the other hand, MDR malignant tumor is the major threat to public health. In this context, natural AMPs have been considered as promising strategy for these two disease threats because of the lower potency of resistance for bacteria and tumor toward AMPs (Felício et al., 2017; Mishra et al., 2017). Owing to typical non-receptor-mediated membrane mechanisms (Sveinbjornsson et al., 2017), anoplin possesses more particular superiority than do conventional antibiotics and chemotherapeutics. In addition, anoplin offers great advantages with simple structure and broad-spectrum activity, especially nonhemolytic toxicity. With the anoplin in hand, it is more convenient to obtain lead compounds for potential translational research. We hope that anoplin and its derivatives will inspire the advancement of AMPs as a novel class of drug candidates.

## Author Contributions

YW and RH wrote the manuscript. J-MJ and L-JZ contributed to the creation of most figures in this manuscript. HZ and H-ZC contributed to the editing of this manuscript. L-LC and XL revised and edited the final manuscript. All authors contributed to reference collection, selection, and final proof.

## Conflict of Interest

The authors declare that the research was conducted in the absence of any commercial or financial relationships that could be construed as a potential conflict of interest.

## References

[B1] Argiolas A. Pisano J. J. (1985). Bombolitins, a new class of mast cell degranulating peptides from the venom of the bumblebee Megabombus pennsylvanicus. J. Biol. Chem. 260, 1437–1444. 10.1016/0196-9781(85)90410-3 2578459

[B2] Arifuzzaman M. Mobley Y. R. Choi H. W. Bist P. Salinas C. A. Brown Z. D.. (2019). MRGPR-mediated activation of local mast cells clears cutaneous bacterial infection and protects against reinfection. Sci. Adv. 5:eaav0216. 10.1126/sciadv.aav0216 30613778 PMC6314830

[B3] Aronson M. R. Simonson A. W. Orchard L. M. Llinas M. Medina S. H. (2018). Lipopeptisomes: anticancer peptide-assembled particles for fusolytic oncotherapy. Acta. Biomater. 80, 269–277. 10.1016/j.actbio.2018.09.025 30240951

[B4] Avrahami D. Shai Y. (2002). Conjugation of a magainin analogue with lipophilic acids controls hydrophobicity, solution assembly, and cell selectivity. Biochemistry 41, 2254–2263. 10.1021/bi011549t 11841217

[B5] Barz B. Liao Q. H. Strodel B. (2018). Pathways of amyloid-β aggregation depend on oligomer shape. J. Am. Chem. Soc. 140, 319–327. 10.1021/jacs.7b10343 29235346

[B6] Braun P. Heijne G. V. (1999). The aromatic residues Trp and Phe have different effects on the positioning of a transmembrane helix in the microsomal membrane. Biochemistry 38, 9778–9782. 10.1021/bi990923a 10423258

[B7] Cabrera M. P. D. S. Arcisio-Miranda M. Costa L. C. D. Souza B. M. D. Costa S. T. B. Palma M. S.. (2009). Interactions of mast cell degranulating peptides with model membranes: a comparative biophysical study. Arch. Biochem. Biophys. 486, 1–11. 10.1016/j.abb.2009.03.009 19328184

[B8] Cabrera M. P. D. S. Arcisio-Miranda M. Costa S. T. B. Konno K. Ruggiero J. R. Procopio J.. (2008). Study of the mechanism of action of anoplin, a helical antimicrobial decapeptide with ion channel-like activity, and the role of the amidated C-terminus. J. Pept. Sci. 14, 661–669. 10.1002/psc.960 17994639

[B9] Carter V. Underhill A. Baber I. Sylla L. Baby M. Larget-Thiery I.. (2013). Killer bee molecules: antimicrobial peptides as effector molecules to target sporogonic stages of Plasmodium. PLoS Pathog. 9:e1003790. 10.1371/journal.ppat.1003790 24278025 PMC3836994

[B10] Carvajal L. A. Neriah D. B. Senecal A. Benard L. Thiruthuvanathan V. Yatsenko T.. (2018). Dual inhibition of MDMX and MDM2 as a therapeutic strategy in leukemia. Sci. Transl. Med. 10:eaao3003. 10.1126/scitranslmed.aao3003 29643228 PMC6130841

[B11] Chamorro C. Boerman M. A. Arnusch C. J. Breukink E. Pieters R. J. (2012). Enhancing membrane disruption by targeting and multivalent presentation of antimicrobial peptides. Biochim. Biophys. Acta 1818, 2171–2174. 10.1016/j.bbamem.2012.04.004 22525599

[B12] Chen Y. Q. Liang J. J. Li T. Lin P. Zhao Y. B. Wu C. L. (2019). Interchain doubly-bridged α-helical peptides for the development of protein binders. Chin. Chem. Lett. 30, 924–928. 10.1016/j.cclet.2019.02.013

[B13] Chen Y. Q. Yang C. Q. Li T. Zhang M. Liu Y. Gauthier M. A.. (2015). The interplay of disulfide bonds, α-helicity, and hydrophobic interactions leads to ultrahigh proteolytic stability of peptides. Biomacromolecules 16, 2347–2355. 10.1021/acs.biomac.5b00567 26156023

[B14] Chen Y. X. Mant C. T. Farmer S. W. Hancock R. E. W. Vasil M. L. Hodges R. S. (2005). Rational design of α-helical antimicrobial peptides with enhanced activities and specificity/therapeutic index. J. Biol. Chem. 280, 12316–12329. 10.1074/jbc.M413406200 15677462 PMC1393284

[B15] Chionis K. Krikorian D. Koukkou A. I. Sakarellos-Daitsiotis M. Panou-Pomonis E. (2016). Synthesis and biological activity of lipophilic analogs of the cationic antimicrobial active peptide anoplin. J. Pept. Sci. 22, 731–736. 10.1002/psc.2939 27862650

[B16] Cong Y. Ji L. Gao Y. J. Liu F. H. Cheng D. B. Hu Z. Y.. (2019). Microenvironment-induced in situ self-assembly of polymer-peptide conjugates that attack solid tumors deeply. Angew. Chem. Int. Ed. 58, 4632–4637. 10.1002/anie.201900135 30695128

[B17] Da Silva A. M. B. Silva-Gonçalves L. C. Oliveira F. A. Arcisio-Miranda M. (2018). Pro-necrotic activity of cationic mastoparan peptides in human glioblastoma multiforme cells via membranolytic action. Mol. Neurobiol. 55, 5490–5504. 10.1007/s12035-017-0782-1 28965321

[B18] Donaldson L. W. Skrynnikov N. R. Choy W. Y. Muhandiram D. R. Sarkar B. Forman-Kay J. D.. (2001). Structural characterization of proteins with an attached ATCUN motif by paramagnetic relaxation enhancement NMR spectroscopy. J. Am. Chem. Soc. 123, 9843–9847. 10.1021/ja011241p 11583547

[B19] Du X. B. Li H. Y. Wang X. H. Liu Q. Ni J. Z. Sun H. Z. (2013). Kinetics and thermodynamics of metal binding to the N-terminus of a human copper transporter, hCTR1. Chem. Commun. 49, 9134–9136. 10.1039/c3cc45360j 23962988

[B20] Fang F. C. (2004). Antimicrobial reactive oxygen and nitrogen species: concepts and controversies. Nat. Rev. Microbiol. 2, 820–832. 10.1038/nrmicro1004 15378046

[B21] Felício M. R. Silva O. N. Gonçalves S. Santos N. C. Franco O. L. (2017). Peptides with dual antimicrobial and anticancer activities. Front. Chem. 5:5. 10.3389/fchem.2017.00005 28271058 PMC5318463

[B22] Harford C. Sarkar B. (1997). Amino terminal Cu(II)- and Ni(II)-binding (ATCUN) motif of proteins and peptides: metal binding, DNA cleavage, and other properties. Acc. Chem. Res. 30, 123–130. 10.1021/ar9501535

[B23] Haug B. E. Stensen W. S. Kalaaji M. Rekdal Ø. Svendsen J. S. (2008). Synthetic antimicrobial peptidomimetics with therapeutic potential. J. Med. Chem. 51, 4306–4314. 10.1021/jm701600a 18570363

[B24] He J. Y. Li C. C. Ding L. Huang Y. Yin X. L. Zhang J. F.. (2019). Tumor targeting strategies of smart fluorescent nanoparticles and their applications in cancer diagnosis and treatment. Adv. Mater. 31:e1902409. 10.1002/adma.201902409 31369176

[B25] He P. P. Li X. D. Wang L. Wang H. (2019). Bispyrene-based self-assembled nanomaterials: in vivo self-assembly, transformation, and biomedical effects. Acc. Chem. Res. 52, 367–378. 10.1021/acs.accounts.8b00398 30653298

[B26] He Y. L. Murby S. Gifford L. Collett A. Warhurst G. Douglas K. T.. (1996). Oral absorption of D-oligopeptides in rats via the paracellular route. Pharm. Res. 13, 1673–1678. 10.1023/A:1016440707092 8956333

[B27] Hisada M. Konno K. Itagaki Y. Naoki H. Nakajima T. (2000). Advantages of using nested collision induced dissociation/post-source decay with matrix-assisted laser desorption/ionization time-of-flight mass spectrometry: sequencing of novel peptides from wasp venom. Rapid Commun. Mass. Spectrom. 14, 1828–1834. 10.1002/1097-0231(20001015)14:19&lt;1828::AID-RCM101&gt;3.0.CO;2-G 11006592

[B28] Hou C. S. Guo L. Q. You L. F. Wang J. R. Lin J. F. Wu W. H. (2014). Molecular cloning and characterization of a venom phospholipase A2 from Apis mellifera spp. J. Entomol. Res. Soc. 16, 55–66.

[B29] Ifrah D. Doisy X. Ryge T. S. Hansen P. R. (2005). Structure-activity relationship study of anoplin. J. Pept. Sci. 11, 113–121. 10.1002/psc.598 15635634

[B30] Jin H. L. Wan C. Zou Z. W. Zhao G. F. Zhang L. L. Geng Y. Y.. (2018). Tumor ablation and therapeutic immunity induction by an injectable peptide hydrogel. ACS Nano 12, 3295–3310. 10.1021/acsnano.7b08148 29558107

[B31] Jindrichova B. Burketova L. Novotna Z. (2014). Novel properties of antimicrobial peptide anoplin. Biochem. Biophys. Res. Commun. 444, 520–524. 10.1016/j.bbrc.2014.01.097 24472551

[B32] Jittikoon J. (2015). Short native α-helical cationic antimicrobial peptides: promising alternative antibiotics. Thai. J. Pharm. Sci. 39, 1–9.

[B33] Kai M. Zhang W. Xie H. Liu L. W. Huang S. J. Li X. (2018). Effects of linker amino acids on the potency and selectivity of dimeric antimicrobial peptides. Chin. Chem. Lett. 29:1163–1166. 10.1016/j.cclet.2018.04.011

[B34] Kamysz E. Sikorska E. Dawgul M. Tyszkowski R. Kamysz W. (2015). Influence of dimerization of lipopeptide Laur-Orn-Orn-Cys–NH_2_ and an N-terminal peptide of human lactoferricin on biological activity. Int. J. Pept. Res. Ther. 21, 39–46. 10.1007/s10989-014-9423-y 25642159 PMC4305368

[B35] Konno K. Hisada M. Fontana R. Lorenzi C. C. Naoki H. Itagaki Y.. (2001). Anoplin, a novel antimicrobial peptide from the venom of the solitary wasp Anoplius samariensis. Biochim. Biophys. Acta 1550, 70–80. 10.1016/S0167-4838(01)00271-0 11738089

[B36] Le C. F. Yusof M. Y. M. Hassan H. Sekaran S. D. (2015). In vitro properties of designed antimicrobial peptides that exhibit potent antipneumococcal activity and produces synergism in combination with penicillin. Sci. Rep. 5:9761. 10.1038/srep09761 25985150 PMC4434909

[B37] Leung B. O. Hitchcock A. P. Won A. Ianoul A. Scholl A. (2011). Imaging interactions of cationic antimicrobial peptides with model lipid monolayers using X-ray spectromicroscopy. Eur. Biophys. J. 40, 805–810. 10.1007/s00249-011-0690-7 21380600

[B38] Li G. Z. Lei Q. F. Wang F. Deng D. S. Wang S. P. Tian L. L.. (2019). Fluorinated polymer mediated transmucosal peptide delivery for intravesical instillation therapy of bladder cancer. Small 15:e1900936. 10.1002/smll.201900936 31074941

[B39] Libardo M. D. Cervantes J. L. Salazar J. C. Angeles-Boza A. M. (2014). Improved bioactivity of antimicrobial peptides by addition of amino-terminal copper and nickel (ATCUN) binding motifs. ChemMedChem 9, 1892–1901. 10.1002/cmdc.201402033 24803240 PMC4440792

[B40] Libardo M. D. Nagella S. Lugo A. Pierce S. Angeles-Boza A. M. (2015). Copper-binding tripeptide motif increases potency of the antimicrobial peptide Anoplin via Reactive Oxygen Species generation. Biochem. Biophys. Res. Commun. 456, 446–451. 10.1016/j.bbrc.2014.11.104 25482446

[B41] Liu B. J. Huang H. F. Yang Z. B. Liu B. Y. Gou S. H. Zhong C.. (2017). Design of novel antimicrobial peptide dimer analogues with enhanced antimicrobial activity *in vitro* and *in vivo* by intermolecular triazole bridge strategy. Peptides 88, 115–125. 10.1016/j.peptides.2016.12.016 28040477

[B42] Liu S. P. Zhou L. Lakshminarayanan R. Beuerman R. W. (2010). Multivalent antimicrobial peptides as therapeutics: design principles and structural diversities. Int. J. Pept. Res. Ther. 16, 199–213. 10.1007/s10989-010-9230-z 20835389 PMC2931633

[B43] Liu Y. F. Xia X. Xu L. Wang Y. Z. (2013). Design of hybrid β-hairpin peptides with enhanced cell specificity and potent anti-inflammatory activity. Biomaterials 34, 237–250. 10.1016/j.biomaterials.2012.09.032 23046754

[B44] Meinike K. Hansen P. R. (2009). Peptoid analogues of anoplin show antibacterial activity. Protein Pept. Lett. 16, 1006–1011. 10.2174/092986609789055421 19799550

[B45] Mishra B. Reiling S. Zarena D. Wang G. (2017). Host defense antimicrobial peptides as antibiotics: design and application strategies. Curr. Opin. Chem. Biol. 38, 87–96. 10.1016/j.cbpa.2017.03.014 28399505 PMC5494204

[B46] Moellering R. E. Cornejo M. Davis T. N. Del B. C. Aster J. C. Blacklow S. C.. (2009). Direct inhibition of the NOTCH transcription factor complex. Nature 462, 182–188. 10.1038/nature08543 19907488 PMC2951323

[B47] Monincová L. Buděšínský M. Slaninová J. Hovorka O. Cvačka J. Voburka Z.. (2010). Novel antimicrobial peptides from the venom of the eusocial bee Halictus sexcinctus (Hymenoptera: Halictidae) and their analogs. Amino Acids 39, 763–775. 10.1007/s00726-010-0519-1 20198492

[B48] Monteiro M. C. Romão P. R. Soares A. M. (2009). Pharmacological perspectives of wasp venom. Protein Pept. Lett. 16, 944–952. 10.2174/092986609788923275 19689421

[B49] Munk J. K. Ritz C. Fliedner F. P. Frimodt-Moller N. Hansen P. R. (2014). Novel method to identify the optimal antimicrobial peptide in a combination matrix, using anoplin as an example. Antimicrob. Agents Chemother. 58, 1063–1070. 10.1128/AAC.02369-13 24277042 PMC3910807

[B50] Munk J. K. Uggerhoj L. E. Poulsen T. J. Frimodt-Moller N. Wimmer R. Nyberg N. T.. (2013). Synthetic analogs of anoplin show improved antimicrobial activities. J. Pept. Sci. 19, 669–675. 10.1002/psc.2548 24019229

[B51] Ng S. Y. Yoshida N. Christie A. L. Ghandi M. Dharia N. V. Dempster J.. (2018). Targetable vulnerabilities in T- and NK-cell lymphomas identified through preclinical models. Nat. Commun. 9:2024. 10.1038/s41467-018-04356-9 29789628 PMC5964252

[B52] Pripotnev S. Won A. Ianoul A. (2010). The effects of L- to D-isomerization and C-terminus deamidation on the secondary structure of antimicrobial peptide Anoplin in aqueous and membrane mimicking environment. J. Raman Spectrosc. 41, 1355–1359. 10.1002/jrs.2608

[B53] Sahariah P. Sorensen K. K. Hjalmarsdottir M. A. Sigurjonsson O. E. Jensen K. J. Masson M.. (2015). Antimicrobial peptide shows enhanced activity and reduced toxicity upon grafting to chitosan polymers. Chem. Commun. 51, 11611–11614. 10.1039/C5CC04010H 26096124

[B54] Salas R. L. Garcia J. Miranda A. Rivera W. L. Nellas R. B. Sabido P. (2018). Effects of truncation of the peptide chain on the secondary structure and bioactivities of palmitoylated anoplin. Peptides 104, 7–14. 10.1016/j.peptides.2018.03.019 29614317

[B55] Schafmeister C. E. Julia Po A. Verdine G. L. (2000). An all-hydrocarbon cross-linking system for enhancing the helicity and metabolic stability of peptides. J. Am. Chem. Soc. 122, 5891–5892. 10.1021/ja000563a

[B56] Schmidt J. O. (1982). Biochemistry of insect venoms. Ann. Rev. Entomol. 27, 339–368. 10.1146/annurev.en.27.010182.002011 7044266

[B57] Schmitt M. A. Weisblum B. Gellman S. H. (2007). Interplay among folding, sequence, and lipophilicity in the antibacterial and hemolytic activities of alpha/beta-peptides. J. Am. Chem. Soc. 129, 417–428. 10.1021/ja0666553 17212422

[B58] Slootweg J. C. van Schaik T. B. Quarles V. U. H. Breukink E. Liskamp R. M. Rijkers D. T. (2013). Improving the biological activity of the antimicrobial peptide anoplin by membrane anchoring through a lipophilic amino acid derivative. Bioorg. Med. Chem. Lett. 23, 3749–3752. 10.1016/j.bmcl.2013.05.002 23719232

[B59] Sveinbjornsson B. Camilio K. A. Haug B. E. Rekdal Ø. (2017). LTX-315: a first-in-class oncolytic peptide that reprograms the tumor microenvironment. Future Med. Chem. 9, 1339–1344. 10.4155/fmc-2017-0088 28490192

[B60] Syed H. Tauseef M. Ahmad Z. (2018). A connection between antimicrobial properties of venom peptides and microbial ATP synthase. Int. J. Biol. Macromol. 119, 23–31. 10.1016/j.ijbiomac.2018.07.146 30053390

[B61] Taft C. S. Selitrennikoff C. P. (1990). Cilofungin inhibition of (1-3)-β-glucan synthase: the lipophilic side chain is essential for inhibition of enzyme activity. J. Antibiot. 43, 433–437. 10.7164/antibiotics.43.433 2141016

[B62] Tsai I. H. Wang Y. M. Cheng A. C. Starkov V. Osipov A. Nikitin I.. (2011). cDNA cloning, structural, and functional analyses of venom phospholipases A_2_ and a Kunitz-type protease inhibitor from steppe viper Vipera ursinii renardi. Toxicon 57, 332–341. 10.1016/j.toxicon.2010.12.012 21185324

[B63] Uggerhoj L. E. Poulsen T. J. Munk J. K. Fredborg M. Sondergaard T. E. Frimodt-Moller N.. (2015). Rational design of alpha-helical antimicrobial peptides: do's and don'ts. Chembiochem 16, 242–253. 10.1002/cbic.201402581 25530580

[B64] Walensky L. D. Bird G. H. (2014). Hydrocarbon-stapled peptides:principles, practice,and progress: miniperspective. J. Med. Chem. 57, 6275–6288. 10.1021/jm4011675 24601557 PMC4136684

[B65] Walensky L. D. Kung A. L. Escher I. Malia T. J. Barbuto S. Wright R. D.. (2004). Activation of apoptosis in vivo by a hydrocarbon-stapled BH_3_ helix. Science 305, 1466–1470. 10.1126/science.1099191 15353804 PMC1360987

[B66] Wang Y. Chen J. B. Zheng X. Yang X. L. Ma P. P. Cai Y.. (2014). Design of novel analogues of short antimicrobial peptide anoplin with improved antimicrobial activity. J. Pept. Sci. 20, 945–951. 10.1002/psc.2705 25316570

[B67] Weinstock M. T. Jacobsen M. T. Kay M. S. (2014). Synthesis and folding of a mirror-image enzyme reveals ambidextrous chaperone activity. Proc. Natl. Acad. Sci. U. S. A. 111, 11679–11684. 10.1073/pnas.1410900111 25071217 PMC4136631

[B68] Wiradharma N. Khoe U. Hauser C. A. Seow S. V. Zhang S. Yang Y. Y. (2011). Synthetic cationic amphiphilic α-helical peptides as antimicrobial agents. Biomaterials 32, 2204–2212. 10.1016/j.biomaterials.2010.11.054 21168911

[B69] Won A. Khan M. Gustin S. Akpawu A. Seebun D. Avis T. J.. (2011a). Investigating the effects of L- to D-amino acid substitution and deamidation on the activity and membrane interactions of antimicrobial peptide anoplin. Biochim. Biophys. Acta 1808, 1592–1600. 10.1016/j.bbamem.2010.11.010 21078293

[B70] Won A. Pripotnev S. Ruscito A. Ianoul A. (2011b). Effect of point mutations on the secondary structure and membrane interaction of antimicrobial peptide anoplin. J. Phys. Chem. B 115, 2371–2379. 10.1021/jp108343g 21338137

[B71] Wu Y. Han M. F. Liu C. Liu T. Y. Feng Y. F. Zou Y. (2017a). Design, synthesis, and antiproliferative activities of stapled melittin peptides. RSC Adv. 7, 17514–17518. 10.1039/C6RA26427A

[B72] Wu Y. Li Y. H. Li X. Zou Y. Liao H. L. Liu L.. (2017b). A novel peptide stapling strategy enables the retention of ring-closing amino acid side chains for the Wnt/β-catenin signalling pathway. Chem. Sci. 8, 7368–7373. 10.1039/C7SC02420G 29163887 PMC5672839

[B73] Wuo M. G. Hong S. H. Singh A. Arora P. S. (2018). Synthetic control of tertiary helical structures in short peptides. J. Am. Chem. Soc. 140, 16284–16290. 10.1021/jacs.8b10082 30395711 PMC7768809

[B74] Wuo M. G. Mahon A. B. Arora P. S. (2015). An effective strategy for stabilizing minimal coiled coil mimetics. J. Am. Chem. Soc. 137, 11618–11621. 10.1021/jacs.5b05525 26340721 PMC4577959

[B75] Yan H. L. Chen W. L. Chen L. L. Ren L. (2011). A novel bioactive peptide with myotropic activity from wasp venoms. Chin. J. Nat. Med. 9, 317–320. 10.1016/S1875-5364(11)60069-1

[B76] Zhong C. Gou S. H. Liu T. Q. Zhu Y. W. Zhu N. Y. Liu H.. (2020a). Study on the effects of different dimerization positions on biological activity of partial d-Amino acid substitution analogues of Anoplin. Microb. Pathog. 139:103871. 10.1016/j.micpath.2019.103871 31733278

[B77] Zhong C. Liu T. Q. Gou S. H. He Y. T. Zhu N. Y. Zhu Y. W.. (2019). Design and synthesis of new N-terminal fatty acid modified-antimicrobial peptide analogues with potent *in vitro* biological activity. Eur. J. Med. Chem. 182:111636. 10.1016/j.ejmech.2019.111636 31466017

[B78] Zhong C. Zhu N. Y. Zhu Y. W. Liu T. Q. Gou S. H. Xie J. Q.. (2020b). Antimicrobial peptides conjugated with fatty acids on the side chain of D-amino acid promises antimicrobial potency against multidrug-resistant bacteria. Eur. J. Pharm. Sci. 141:105123. 10.1016/j.ejps.2019.105123 31676352

[B79] Zhu L. N. Fu C. Y. Zhang S. F. Chen W. Jin Y. T. Zhao F. K. (2013). Novel cytotoxic exhibition mode of antimicrobial peptide anoplin in MEL cells, the cell line of murine Friend leukemia virus-induced leukemic cells. J. Pept. Sci. 19, 566–574. 10.1002/psc.2533 23873700

